# Impact of weight on daily activities questionnaire in patients with overweight or obesity: Psychometric evaluation using data from the OASIS 1 trial

**DOI:** 10.1111/cob.70015

**Published:** 2025-06-01

**Authors:** Lisa von Huth Smith, Diane Whalley, Stuart Yarr, Jonathan Comins, Sheri E. Fehnel

**Affiliations:** ^1^ Novo Nordisk A/S Søborg Denmark; ^2^ Patient‐Centered Outcomes Assessment RTI Health Solutions, Towers Business Park Manchester UK; ^3^ Patient‐Centered Outcomes Assessment RTI Health Solutions Research Triangle Park North Carolina USA

**Keywords:** daily activities, patient‐reported outcome, psychometric, semaglutide, weight loss

## Abstract

The Impact of Weight on Daily Activities Questionnaire (IWDAQ) is a patient‐reported outcome measure that uses a novel, adaptive design to assess the limitations in daily activities that are most important to individuals attempting to lose weight. During the first round of completing the IWDAQ, respondents are presented with 18 everyday activities that can be limited by excess weight and asked to choose the three activities they would most like to see improve with weight loss. They are then asked to rate the degree of limitation they experience with these three activities at baseline and at follow‐up assessments. Using data from a weight‐management clinical trial (OASIS 1, NCT05035095), we evaluated the IWDAQ's measurement properties, determined optimal scoring, and estimated thresholds of meaningful within‐patient change. Our analyses demonstrated that the IWDAQ Composite Score offers a reliable and valid personalized measure of limitations in daily activities due to excess weight. The adaptive design of the IWDAQ ensures the patient‐centricity of the measure, thereby complementing existing measures of functioning in the context of weight‐management clinical trials. Evaluations using data from additional studies would be valuable in extending the psychometric evidence for the IWDAQ.

**What is already known about this subject**

- Obesity (body mass index ≥30 kg/m^2^) is a global health concern that can impair patients' physical functioning, activities of daily living, and health‐related quality of life (HRQOL).
- The Impact of Weight on Daily Activities Questionnaire (IWDAQ)—comprising 18 activities—is a novel, adaptive patient‐reported outcome measure designed to capture changes in patient‐relevant activity limitations due to excess weight.
- The IWDAQ was developed in accordance with current regulatory standards, which centre on incorporating the patient voice through qualitative research.

**What this study adds**

- This study evaluated the measurement properties of the IWDAQ using data from OASIS 1 (NCT05035095), a phase 3a study of oral, once‐daily semaglutide 50 mg for the treatment of overweight or obesity.
- Analyses determined optimal scoring of the IWDAQ, supported the psychometric properties of the measure within a composite model framework, and estimated thresholds for meaningful within‐patient change in the IWDAQ Composite Score.
- Our findings demonstrate that the IWDAQ is a reliable and valid personalized measure of activity limitations due to excess weight and that it can complement measures of patient functioning and HRQOL commonly used in weight‐management clinical trials to provide a more comprehensive assessment of treatment benefit.

## 1 INTRODUCTION

Obesity is a complex, common, and chronic disease characterized by a body mass index (BMI) ≥30 kg/m^2^.^1^, ^2^ Since 1990, the incidence of adult obesity has roughly doubled worldwide, with the condition affecting 890 million adults in 2022 alone.^1^ Excess body weight contributed to approximately 5 million deaths globally in 2019,^1^ making obesity a significant public health concern. Obesity is associated with significant decrements in patients' health and functional status, which cause considerable medical and societal burden.^2^, ^3^ The impacts of obesity vary based on environmental, biologic, and socioeconomic factors, but risks for preventable health complications such as cardiovascular disease, type 2 diabetes, and some cancers increase as BMI increases.^1^, ^2^ Moreover, people with obesity experience weight bias, stigma, limitations in physical functioning, reduced ability to participate in activities of daily living, and decreased health‐related quality of life (HRQOL).^2^, ^4^, ^5^

Sustained weight loss may improve obesity‐related comorbidities, functional status, and HRQOL.^4^, ^6^, ^7^ There is a need for measures evaluating those weight‐management goals and outcomes that are important from the perspective of patients undergoing treatment for overweight or obesity. The Impact of Weight on Daily Activities Questionnaire (IWDAQ) is the first patient‐reported outcome (PRO) measure that evaluates the limitations in daily activities due to excess weight that clinical trial participants would most like to improve with weight loss (e.g., exercise, household tasks, physical activities at work, taking care of children).^4^ Designed to complement functional assessments frequently used in obesity clinical trials (e.g., the Impact of Weight on Quality of Life–Lite Clinical Trials Version [IWQOL‐Lite‐CT]^8^, ^9^, ^10^ and the SF‐36v2 Health Survey [SF‐36v2]^11^), the IWDAQ was developed in accordance with US Food and Drug Administration (FDA) PRO guidance,^12^ refined through qualitative hybrid (concept elicitation and cognitive debriefing) research, and uses an adaptive questionnaire design to measure the effects of weight loss.^4^ The IWDAQ includes a list of 18 activities that can be impacted by excess weight, from which respondents choose the three they would most like to see improve with weight loss. The extent to which these activities are limited is then rated at baseline and monitored over time. As such, the IWDAQ is intended to be a personalized assessment of change in the day‐to‐day activities that matter most to an individual patient.

The objective of this analysis was to explore the measurement properties of the IWDAQ using data from OASIS 1 (NCT05035095),^13^ a phase 3a study evaluating the efficacy and safety of oral semaglutide 50 mg taken once per day for the treatment of overweight or obesity. Our specific aims were to determine the optimal scoring, evaluate the psychometric properties of the measure within a composite model framework, and estimate thresholds of meaningful within‐patient change (MWPC) in scores.

## 2 METHODS

### 2.1 Study design

OASIS 1 (NCT05035095)^13^ was a phase 3a, randomized, double‐blind, placebo‐controlled clinical trial that assessed the efficacy, safety, and tolerability of oral semaglutide in patients with overweight or obesity. Trial design and eligibility criteria were described previously.^13^ Briefly, 667 adults with BMI ≥30.0 kg/m^2^ (or ≥27.0 kg/m^2^ with ≥1 body weight‐related comorbidity) were randomly assigned to receive 50 mg semaglutide or placebo once daily for 68 weeks as an adjunct to lifestyle intervention.

The primary efficacy endpoints in OASIS 1 (relative change in body weight and the achievement of ≥5% reduction in body weight) were assessed as change from baseline at week 68, and this timepoint was used in the current analyses to evaluate responsiveness and estimate MWPC thresholds for the IWDAQ. Cross‐sectional properties, including score distribution and construct validity, were also evaluated using data at week 68, as well as at baseline. Additionally, to assess the stability and reproducibility of IWDAQ scores, data at week 52 and week 68 were used to evaluate test–retest reliability. The analysis sample included all participants in the full analysis set (all randomized subjects) who completed the IWDAQ at baseline.

### 2.2 PRO measures

The IWDAQ^4^ is a newly developed PRO measure designed to assess the impact of weight change on daily activity limitations commonly experienced by people with excess weight. The content was developed from a targeted review of the literature, secondary analysis of qualitative data collected during the development of the IWQOL‐Lite‐CT^8^ and the Weight‐Related Signs and Symptoms Measure (WRSSM),^14^ and interviews with three clinical experts in obesity. Content validity was confirmed through qualitative hybrid (concept elicitation and cognitive debriefing) interviews with 46 individuals with a BMI ≥30 kg/m^2^ in the United States and United Kingdom.^4^ The measure uses an adaptive design and includes a list of 18 activities that may be impacted by excess weight (Table S1). At baseline, participants first select three activities from the list that they most want to see improve with weight loss (step 1). Participants then rate the current limitation they experience in each of these three activities using a 5‐point response scale (1 = Not at all, 2 = A little, 3 = A moderate amount, 4 = A great deal, 5 = An extreme amount) (step 2). At each follow‐up assessment, participants rate the degree of limitation in the same three activities using the same 5‐point response scale. The IWDAQ Composite Score is computed as the sum of the three activity scores. Scores range from 3 to 15, with higher scores indicating greater activity limitations for the participant.

Responses to other study measures were used in the current analyses to assess the measurement properties of the IWDAQ, as appropriate. These supporting measures, summarized in Table S1, included several PGIS and Patient Global Impression of Change (PGI‐C) items, the IWQOL‐Lite‐CT, and the acute version of the SF‐36v2. All PRO measures, including the IWDAQ, were completed electronically by OASIS 1 participants using a tablet device.

### 2.3 Analysis methods

All analyses were blinded to treatment arm and were performed using SAS Studio. The data were analysed as observed, with no imputation for missing visit‐level data. Participants in OASIS 1 were required to complete all three IWDAQ activity ratings, and thus, there were no missing activity‐level responses.

To evaluate the distributional properties of the IWDAQ, the number of participants who selected each of the IWDAQ activities at baseline as one of the three activities they most wanted to see improve with weight loss was evaluated, as was the frequency with which different combinations of activities were selected by participants. Descriptive statistics (including by BMI categories [≥27 to <30, ≥30 to <35, ≥35 to <40, and ≥40]), response distributions, and percentages were evaluated for individual activities. The IWDAQ Composite Score was evaluated using descriptive statistics, and floor and ceiling effects were defined as >15% of participants obtaining the worst or best score, respectively.^15^ In addition, analyses using the Spearman‐Brown prophecy formula^16^, ^17^ were conducted to determine the number of activity scores needed to achieve a sum score with good reliability (i.e., intraclass correlation coefficient [ICC] ≥0.75^18^) for the computation of the IWDAQ Composite Score. Test–retest reliability^19^ was evaluated using IWDAQ activity and composite scores at week 52 (test) and week 68 (retest) for the overall sample, over which time participants' status was expected to remain stable; however, to account for any change between these time points, participants with less than 5% change in body weight and no change on a PGI‐S (Necessary Activities and Desired Activities) were used as stable subsamples over this period. For IWDAQ activity scores, the weighted kappa coefficient was computed; coefficients 0.41–0.60 indicate moderate agreement, 0.61–0.80 indicate substantial agreement, and 0.81–1.00 indicate almost perfect agreement.^20^ For IWDAQ composite scores, ICCs were computed using two‐way mixed‐effects (random subject × fixed time) analysis of variance (ANOVA) models with absolute agreement for single measures.^19^, ^21^ It is recommended that ICCs be at least 0.70 to demonstrate acceptable levels of reliability.^22^

To support construct validity, correlational analysis was conducted to evaluate the associations between the IWDAQ Composite Score and scores on other supporting measures at baseline and week 68. Pearson correlations were computed with IWQOL‐Lite‐CT composite scores, SF‐36v2 subscale scores, and body weight; polyserial correlations were computed with PGI‐S item scores and BMI categories. On the basis of guidelines adapted from Cohen^23^ and Hinkle et al.,^24^ correlations <0.30 were considered negligible or low, ≥0.30 to <0.70 were considered moderate, ≥0.70 to <0.90 were considered high, and ≥0.90 were considered very high. The magnitude and direction of the correlation coefficients were evaluated against a priori hypotheses to demonstrate convergent validity (measures addressing similar or related constructs) and discriminant validity (measures addressing more disparate constructs). Positive, moderate correlations were expected with PGI‐S Necessary Activities and PGI‐S Desired Activities, and negative, moderate correlations were expected with PGI‐S Physical Function, PGI‐S Mental Health, IWQOL‐Lite‐CT scores, and SF‐36v2 acute Physical Component Summary (PCS) and Mental Component Summary (MCS) scores. In addition, the ability of the IWDAQ Composite Score to discriminate between groups was evaluated using an ANOVA to compare mean scores across PGI‐S (Necessary Activities, Desired Activities, Physical Function, and Mental Health) and BMI category subgroups.

Responsiveness of the IWDAQ Composite Score was evaluated using change from baseline to week 68. ANOVA models were used to examine differences in mean change in the IWDAQ Composite Score between subgroups of change based on PGI‐S, PGI‐C, and body weight. Pearson correlations were computed between change from baseline in the IWDAQ Composite Score and change in scores on supporting measures. At least moderate correlations (|r| ≥ 0.30) were hypothesized with changes based on PGI‐S, PGI‐C, IWQOL‐Lite‐CT total and composite scores, SF‐36v2 acute PCS and MCS scores, and body weight.

Finally, following established guidance,^12^, ^25^, ^26^, ^27^ MWPC thresholds were estimated from the median change from baseline in the IWDAQ Composite Score among subgroups of participants with defined levels of change on the PGI‐S and PGI‐C items. Appropriateness of these measures as anchors for change in the IWDAQ Composite Score was determined by correlations of change of at least 0.37.^28^ A one‐category improvement on the PGI‐S Desired Activities was the predefined primary anchor, consistent with findings from qualitative interviews conducted during development of the IWDAQ, in which most participants (>80%) indicated that this level of improvement on the PGI‐S would be meaningful. Supportive anchors included a two‐category improvement on the PGI‐S Desired Activities, one‐category and two‐category improvements on the PGI‐S Necessary Activities, and a change to ‘somewhat less limited now’ on PGI‐C Necessary Activities and PGI‐C Desired Activities. Distribution‐based statistics (the half–standard deviation [SD] and the standard error of measurement, computed as the SD multiplied by the square root of 1 minus the test–retest reliability ICC) were computed to provide information about measurement variability and used to inform the lower bound for MWPC thresholds.

## 3 RESULTS

### 3.1 Baseline characteristics of the study population

The IWDAQ was implemented in each country in OASIS 1 as the relevant translated version(s) became available. Of the 667 participants in OASIS 1, IWDAQ data were available for 366 participants (Table 1). The mean (SD) age of the sample was 50.6 (13.16) years, and most participants were female (74%), were White (71%), and did not report Hispanic or Latino ethnicity (81%). The mean (SD) BMI was 37.4 (6.10); 20 (5.5%) participants had a BMI of ≥27 to <30, 124 (33.9%) had a BMI of ≥30 to <35, 123 (33.6%) had a BMI of ≥35 to <40, and 99 (27.0%) had a BMI ≥40. Descriptive statistics for IWQOL‐Lite‐CT scores, SF‐36v2 acute scores, PGI‐S item scores, BMI, and body weight at baseline and week 68 are summarized in Table S2.

**TABLE 1:** Participant characteristics at baseline.

Participant characteristic	OASIS 1 psychometric analysis sample (*N* = 366)
Age (years)	
Mean (SD), median	50.6 (13.16), 51.0
Min, max	18.0, 81.0
Sex, *n* (%)	
Female	269 (73.5)
Male	97 (26.5)
Weight (kg)	
Mean (SD), median	104.7 (20.41), 100.8
Min, max	66.0, 186.5
BMI	
Mean (SD), median	37.4 (6.10), 36.2
Min, max	27.0, 61.6
BMI categories, *n* (%)	
≥27 to <30	20 (5.5)
≥30 to <35	124 (33.9)
≥35 to <40	123 (33.6)
≥40	99 (27.0)
Race, *n* (%)	
Asian	22 (6.0)
Black or African American	42 (11.5)
Native Hawaiian or Other Pacific Islander	2 (0.5)
White	258 (70.5)
Other	3 (0.8)
Not reported	39 (10.7)
Ethnicity, *n* (%)	
Hispanic or Latino	25 (6.8)
Not Hispanic or Latino	298 (81.4)
Not reported	43 (11.7)

Abbreviations: BMI, body mass index; SD, standard deviation.

### 3.2 IWDAQ score distributions

The IWDAQ Composite Score decreased from a mean of 8.73 at baseline to a mean of 5.58 at week 52 and a mean of 5.39 at week 68. There was no evidence of problematic floor (worst status) or ceiling (best status) effects at baseline, with less than 15% of participants obtaining the maximum possible score (i.e., 15) or the minimum possible score (i.e., 3), thus allowing scores to demonstrate both improvement and worsening from baseline.

**FIGURE 1:**
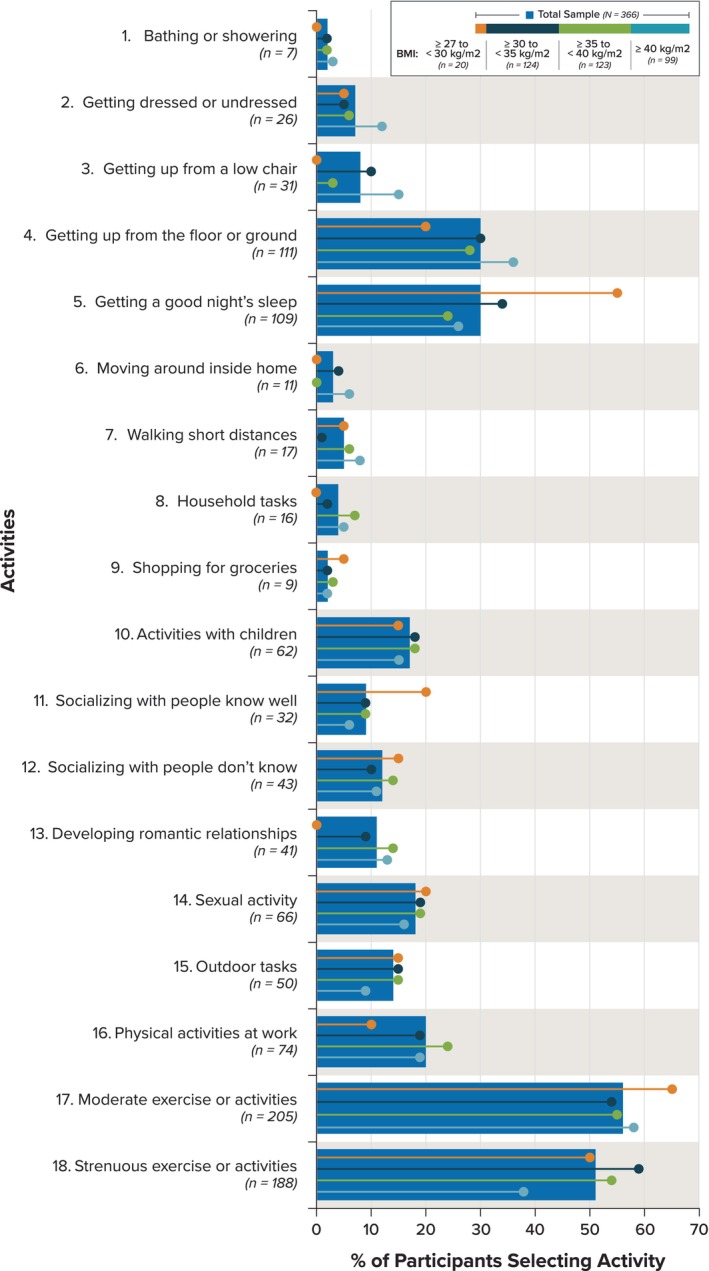
Percentage of participants selecting each activity at baseline for the total sample and by BMI categories.

Across the 366 participants, there were a total of 152 different combinations of activities selected by participants. Figure 1 presents the percentage of participants selecting each of the 18 IWDAQ activities at baseline for the total sample and by BMI categories. Among the total sample, moderate activities (activity 17) and strenuous activities (activity 18) were selected most frequently (56% and 51%, respectively), followed by getting up from the floor or ground (activity 4; 30%) and getting a good night's sleep (activity 5; 30%). The least frequently selected activities were bathing or showering (activity 1; 2%), shopping for groceries (activity 9; 2%), moving around inside the home (activity 6; 3%), household tasks (activity 8; 4%), and walking short distances (activity 7; 5%). Compared with the total sample, more participants with a BMI ≥40 selected basic activities such as getting dressed or undressed (12% vs. 7% in the total sample), getting up from a low chair (15% vs. 8% in the total sample), and getting up from the floor or ground (36% vs. 30% in the total sample). In contrast, proportionately fewer participants with a BMI of ≥27 to <30 selected these types of activities and were instead more likely to select activities such as getting a good night's sleep (55% vs. 30% in the total sample), socializing with people they know well (20% vs. 9% in the total sample), and moderate exercise or activities (65% vs. 56% in the total sample).

Descriptive statistics (mean, SD, and median) and response distributions for the individual IWDAQ activities at baseline and at week 68 are presented in Table S3. At baseline, mean activity scores (possible range 1–5) ranged from 2.1 (bathing or showering) to 3.2 (getting up from the floor or ground, developing romantic relationships, sexual activity, and strenuous exercise or activities). Ceiling effects (defined as more than 40% of participants responding with the best response, ‘not at all’, at baseline) were observed for one activity (shopping for groceries; 44%), although only nine participants selected this activity. There were no floor effects at baseline. At week 68, scores ranged from 1.2 (bathing or showering) to 2.2 (getting up from the floor or ground). As would be expected, more participants obtained the best activity scores at this timepoint.

### 3.3 Scoring analysis

The intended purpose of the IWDAQ as a personalized measure, the diversity in the selection of activities, and the variability in the patterns of associations across activities and with external measures suggest that a composite measurement model is an appropriate representation of the underlying conceptual framework for the IWDAQ. Within the framework, the IWDAQ Composite Score for an individual participant is calculated as the sum of the limitation ratings for the three activities selected by that participant. Because the three activities are selected as the most important to the individual, the three scores are equally weighted in the composite. Using the Spearman‐Brown prophecy formula, it was estimated that 2.3 activity scores at baseline and 1.8 activity scores at week 68 were needed to yield a total score with at least good reliability (i.e., ICC of 0.75). Such results indicate that all three activity scores are needed to maximize the reliability of the IWDAQ Composite Score.

### 3.4 Test–retest reliability

For the IWDAQ Composite Score, the ICCs (95% confidence interval [CI]) were 0.79 (0.74, 0.83) for the overall sample, 0.82 (0.76, 0.86) for participants with <5% change in body weight and no change on PGI‐S Desired Activities, and 0.77 (0.71, 0.82) for participants with <5% change in body weight and no change on PGI‐S Necessary Activities. These values were all well above 0.70 (the accepted minimum standards for multi‐item measures^22^), providing strong support for the reliability of the IWDAQ Composite Score. The weighted kappa coefficients for the individual activity scores between weeks 52 and 68 are shown in Table S4.

### 3.5 Construct validity correlations

Table 2 presents construct validity correlations for the IWDAQ Composite Score at baseline and week 68. The strongest correlations were with the IWQOL‐Lite‐CT total and composite scores (range, *r* = −0.56 to −0.72), the PGI‐S Desired Activities score (*r* = 0.72–0.75), the PGI‐S Necessary Activities score (*r* = 0.58–0.76), and the PGIS‐Physical Function score (*r* = −0.52 to −0.62). The lowest correlations were with the SF36v2 acute MCS (*r* = −0.17 to −0.18), Role Limitations–Emotional (*r* = −0.23 to −0.31), and Mental Health (*r* = −0.26 to −0.27) scores, as well as with body weight (*r* = 0.24–0.44). Validation hypotheses were generally met.

**TABLE 2:** Correlations between the IWDAQ composite score and scores from supporting measures at baseline and week 68 and for change from baseline to week 68.

Supporting measure	IWDAQ composite score correlation coefficient (*n*)		
	Baseline	Week 68	Change from baseline to week 68
IWQOL‐Lite‐CT			
Total	−0.63* (366)	−0.71* (320)	−0.64* (320)
Physical	−0.62* (366)	−0.72* (320)	−0.60* (320)
Physical function	−0.61* (366)	−0.71* (320)	−0.57* (320)
Psychosocial	−0.56* (366)	−0.60* (320)	−0.58* (320)
SF‐36v2 acute			
PCS	−0.44* (366)	−0.59* (320)	−0.50* (320)
MCS	−0.17* (366)	−0.18* (320)	−0.21* (320)
Physical functioning	−0.49* (366)	−0.65* (320)	−0.44* (320)
Role limitations–physical	−0.38* (366)	−0.50* (320)	−0.43* (320)
Bodily pain	−0.34* (366)	−0.47* (320)	−0.38* (320)
General health	−0.30* (366)	−0.43* (320)	−0.37* (320)
Vitality	−0.37* (366)	−0.41* (320)	−0.39* (320)
Social functioning	−0.35* (366)	−0.34* (320)	−0.37* (320)
Role limitations–emotional	−0.23* (366)	−0.31* (320)	−0.28* (320)
Mental health	−0.26* (366)	−0.27* (320)	−0.15* (320)
**PGI‐S**			
PGI‐S desired activities	0.72* (366)	0.75* (319)	0.60* (319)
PGI‐S necessary activities	0.58* (366)	0.76* (320)	0.54* (320)
PGI‐S physical function	−0.52* (365)	−0.62* (320)	−0.40* (320)
PGI‐S mental health	−0.33* (366)	−0.43* (320)	−0.41* (320)
PGI‐C			
PGI‐C desired activities	N/A	N/A	0.45* (319)
PGI‐C necessary activities	N/A	N/A	0.45* (320)
PGI‐C physical function	N/A	N/A	0.48* (320)
PGI‐C mental health	N/A	N/A	0.41* (320)
Weight‐related measures			
BMI category	0.33* (366)	0.56* (320)	0.39* (320)
Body weight	0.24* (366)	0.44* (320)	0.43* (320)
Body weight % change	N/A	N/A	0.42* (320)

*Note*: Gray shading indicates correlations that met validation hypotheses, green shading indicates correlations that were stronger than hypothesized, orange shading indicates correlations that were weaker than hypothesized, and no shading indicates that no validation hypotheses were made. Hypotheses were positive, moderate correlations with the PGI‐S Necessary Activities and PGI‐S Desired Activities, and negative, moderate correlations with PGI‐S Physical Function, PGI‐S Mental Health, IWQOL‐Lite‐CT total and composite scores, and SF‐36v2 acute PCS and MCS scores.

Abbreviations: BMI, body mass index; IWDAQ, Impact of Weight on Daily Activities Questionnaire; IWQOL‐Lite‐CT, Impact of Weight on Quality of Life–Lite Clinical Trials Version; MCS, Mental Component Summary; N/A, not applicable; PCS, Physical Component Summary; PGI‐S, Patient Global Impression of Status; SF‐36v2, Short Form Health Survey‐36, version 2.

* *p* < .05 for H_0_: *ρ* = 0.

### 3.6 Known‐groups analysis

Table 3 presents descriptive statistics for the IWDAQ Composite Score across subgroups defined according to responses to the PGIS items and BMI categories at baseline and week 68. The IWDAQ Composite Score showed the expected pattern across the subgroups; specifically, higher mean scores (indicating greater activity limitations) were observed for subgroups of participants reporting greater limitations in their ability to do the activities they want to do (PGIS Desired Activities) or need to do (PGI‐S Necessary Activities), worse physical functioning (PGI‐S Physical Function), poorer mental health (PGI‐S Mental Health), and higher BMI. Analysis of variance indicated statistically significant differences across the subgroups in all cases (all *p* < .0001; Table 3). Pairwise *t*‐tests showed statistically significant differences (*p* < .05) between all nonadjacent subgroups. There were no differences in the mean IWDAQ Composite Score by age or sex (*p* > .05 in all cases).

**TABLE 3:** Descriptive statistics for the IWDAQ composite score by PGI‐S and BMI subgroups at baseline and week 68.

Supporting measure subgroups	Baseline		Week 68	
	IWDAQ score: mean (SD), median [*n*]	ANOVA, *F*; *p* value	IWDAQ score: mean (SD), median [*n*]	ANOVA, *F*; *p* value
PGI‐S desired activities				
Not at all	6.0 (2.20), 6.0 [74]	83.1; <.0001	4.0 (1.49), 3.0 [174]	101.5; <.0001
A little	8.2 (1.94), 8.0 [147]		6.0 (1.88), 6.0 [99]	
A moderate amount	9.7 (1.76), 10.0 [87]		8.4 (2.50), 8.5 [28]	
A great deal	11.7 (2.30), 12.0 [46]		10.5 (2.36), 11.0 [15]	
An extreme amount	13.3 (1.22), 13.5 [12]		14.3 (0.58), 14.0 [3]	
PGI‐S necessary activities				
Not at all	7.2 (2.48), 7.0 [124]	38.4; <.0001	4.4 (1.65), 4.0 [232]	125.6; <.0001
A little	8.7 (2.31), 9.0 [154]		7.1 (2.21), 7.0 [66]	
A moderate amount	10.4 (1.81), 10.0 [66]		10.5 (2.10), 10.0 [15]	
A great deal	12.6 (2.58), 13.0 [20]		12.9 (1.57), 13.0 [7]	
An extreme amount	14.0 (1.41), 14.0 [2]		− (−), − [0]	
PGI‐S physical function				
Excellent	5.7 (1.99), 6.0 [16]	29.9; <.0001	3.3 (1.23), 3.0 [35]	50.5; <.0001
Very good	7.0 (2.27), 7.0 [58]		4.3 (1.74), 4.0 [95]	
Good	8.2 (2.35), 9.0 [111]		5.5 (2.09), 5.0 [118]	
Fair	9.6 (2.36), 10.0 [138]		6.7 (2.58), 6.0 [56]	
Poor	11.1 (2.80), 11.0 [42]		11.0 (3.16), 11.5 [16]	
PGI‐S mental health				
Excellent	6.5 (2.19), 6.0 [20]	11.1; <.0001	4.1 (1.92), 3.0 [56]	19.1; <.0001
Very good	8.1 (2.76), 8.0 [73]		4.5 (1.93), 4.0 [108]	
Good	8.6 (2.65), 9.0 [141]		6.2 (2.71), 6.0 [94]	
Fair	9.1 (2.46), 9.0 [101]		6.6 (2.66), 6.0 [53]	
Poor	10.9 (2.59), 11.0 [31]		8.9 (4.46), 10.0 [9]	
BMI category				
<27	– [0]	13.3; <.0001	3.9 (1.40), 3.0 [54]	33.5; <.0001
≥27 to <30	7.3 (2.20), 7.0 [20]		4.1 (1.64), 4.0 [51]	
≥30 to <35	8.0 (2.68), 8.0 [124]		4.9 (2.03), 4.0 [95]	
≥35 to <40	8.7 (2.58), 9.0 [123]		6.2 (2.53), 6.0 [69]	
≥40	10.0 (2.62), 10.0 [99]		8.2 (3.21), 8.0 [51]	

Abbreviations: ANOVA, analysis of variance; BMI, body mass index; IWDAQ, Impact of Weight on Daily Activities Questionnaire; PGI‐S, Patient Global Impression of Status; SD, standard deviation.

### 3.7 Responsiveness analysis

Table 2 presents responsiveness correlations between change in the IWDAQ Composite Score from baseline to week 68 and changes in scores from the supporting measures. As expected, the correlations were at least moderate (|*r*| ≥ 0.30) with changes based on PGI‐S, PGI‐C, IWQOL‐Lite‐CT total and composite scores, SF‐36v2 acute PCS scores, and body weight. Only the correlation with change in the SF‐36v2 acute MCS score was lower than hypothesized (−0.21). The strongest associations were with changes in the PGIS Desired Activities (*r* = 0.60), the PGIS Necessary Activities (*r* = 0.54), and the IWQOL‐Lite‐CT composite scores (*r* = −0.57 [Physical Function] to −0.64 [Total]). The weakest associations were with the SF‐36v2 acute MCS (*r* = −0.21), Role Limitations–Emotional (*r* = −0.28), and Mental Health (*r* = −0.15) scores.

Table 4 presents descriptive statistics for the IWDAQ Composite Score across levels of change on PGI‐S, PGI‐C, and body weight from baseline to week 68. Mean change in the IWDAQ Composite Score showed the expected pattern across the defined change groups. Analysis of variance indicated statistically significant differences across the change subgroups in all cases (all *p* < .0001). Pairwise *t*‐tests demonstrated that the mean IWDAQ Composite Scores for the improved subgroups were statistically different (*p* < .05) from the scores for the no change and worsened subgroups in most cases.

**TABLE 4:** Descriptive statistics for change in the IWDAQ composite score from baseline to week 68 by change in PGI‐S, PGI‐C, and percentage change in body weight.

Supporting measure subgroups	IWDAQ change score: mean (SD), median [*n*]	Overall, *F*; *p* value
Change in PGI‐S desired activities		
Improved ≥3 categories	−7.7 (2.81), −8.0 [14]	43.3; <.0001
Improved 2 categories	−5.5 (2.13), −6.0 [48]	
Improved 1 category	−3.7 (2.59), −4.0 [118]	
No change	−2.1 (2.06), −2.0 [112]	
Worsened ≥1 categories	−0.4 (1.99), −1.0 [27]	
Change in PGI‐S necessary activities		
Improved ≥3 categories	−7.8 (2.04), −8.0 [6]	31.2; <.0001
Improved 2 categories	−5.9 (2.25), −6.0 [35]	
Improved 1 category	−3.9 (2.48), −4.0 [128]	
No change	−2.5 (2.50), −3.0 [127]	
Worsened ≥1 categories	−0.1 (2.11), −0.5 [24]	
Change in PGI‐S physical function		
Improved ≥3 categories	−7.4 (3.27), −7.5 [10]	16.7; <.0001
Improved 2 categories	−4.7 (3.05), −5.0 [40]	
Improved 1 category	−3.9 (2.37), −4.0 [110]	
No change	−2.5 (2.50), −2.0 [130]	
Worsened ≥1 categories	−1.7 (3.02), −2.0 [30]	
Change in PGI‐S mental health		
Improved ≥3 categories	−5.6 (3.50), −5.0 [23]	16.0; <.0001
Improved 2 categories	−4.9 (2.69), −4.5 [44]	
Improved 1 category	−3.8 (2.43), −3.5 [92]	
No change	−2.7 (2.48), −3.0 [108]	
Worsened ≥1 categories	−1.6 (2.72), −2.0 [53]	
PGI‐C desired activities		
Much less limited	−4.6 (2.80), −5.0 [124]	16.3; <.0001
Somewhat less limited	−3.7 (2.42), −3.0 [45]	
A little less limited	−2.3 (2.25), −2.0 [39]	
No change	−2.3 (2.55), −2.0 [102]	
A little/somewhat/much more limited	−0.1 (3.14), −1.0 [9]	
PGI‐C necessary activities		
Much less limited	−4.6 (2.66), −4.0 [128]	16.2; <.0001
Somewhat less limited	−3.2 (2.64), −3.0 [47]	
A little less limited	−2.5 (2.63), −2.0 [35]	
No change	−2.3 (2.50), −2.0 [103]	
A little/somewhat/much more limited	0.1 (3.44), −1.0 [7]	
PGI‐C physical function		
Much better	−4.6 (2.86), −4.0 [125]	18.9; <.0001
Moderately better	−3.6 (2.63), −3.0 [66]	
A little better	−2.1 (2.01), −2.0 [44]	
No difference	−2.1 (2.24), −2.0 [78]	
A little/moderately/much worse	0.3 (3.45), −1.0 [7]	
PGI‐C mental health		
Much better	−4.4 (2.91), −4.0 [115]	15.9; <.0001
Moderately better	−4.1 (2.16), −4.0 [63]	
A little better	−2.2 (2.37), −2.0 [46]	
No difference	−2.4 (2.58), −2.0 [86]	
A little/moderately/much worse	0.5 (3.17), 1.0 [10]	
% change in body weight		
≥15% loss	−4.6 (2.78), −4.0 [105]	14.3; <.0001
≥10% to <15% loss	−3.8 (2.84), −3.0 [39]	
≥5% to <10% loss	−3.4 (2.36), −3.0 [47]	
<5% change	−2.2 (2.59), −2.0 [119]	
≥5% gain	−0.7 (2.11), −0.5 [10]	

*Note*: Change is from baseline to week 68; negative change scores indicate improvement.

Abbreviations: IWDAQ, Impact of Weight on Daily Activities Questionnaire; MWPC, meaningful within‐patient change; PGI‐C, Patient Global Impression of Change; PGI‐S, Patient Global Impression of Status; SD, standard deviation.

### 3.8 MWPC thresholds

The responsiveness correlation indicated an acceptable level of association between change in the IWDAQ Composite Score and change in the primary anchor measure, the PGI‐S Desired Activities (*r* = 0.60). This correlation, along with the empirical cumulative distribution function plot for change from baseline to week 68 for the IWDAQ Composite Score for each level of change on PGI‐S Desired Activities (Figure S1), provides support for a one‐category improvement in the PGI‐S Desired Activities serving as an appropriate anchor to determine MWPC for improvement on the IWDAQ Composite Score. Correlations between change in the IWDAQ Composite Score and changes in the additional anchor measures were also adequate (PGIS Necessary Activities, *r* = 0.54; PGIC Desired Activities, *r* = 0.45; PGI‐C Necessary Activities, *r* = 0.45).

Table 5 summarizes the mean, median, and 95% CIs for the IWDAQ Composite Score as it corresponds to the predefined primary anchor and the supporting anchors that were confirmed to be adequate. The mean and median values were similar, and the median values were used to define the threshold estimates to ensure that at least half of the participants at any given anchor level obtained the observed score. The median change in the IWDAQ Composite Score among participants with a one‐category improvement on the PGI‐S Desired Activities (the predefined primary anchor) was −4.0 (95% CI, −4.20 to −3.26). Similar estimates were obtained across participants with different levels of baseline severity, ranging from a median of −2.0 for a single participant responding ‘an extreme amount’ at baseline to −5.0 for 9 participants responding ‘a great deal’. The median estimates for the baseline severity subgroups with the most robust sample sizes (i.e., participants reporting ‘a little’ or ‘a moderate amount’ at baseline) were both −4.0. The primary estimate was also consistent across participants with different baseline BMI; specifically, within the primary anchor group (one‐category improvement on the PGIS Desired Activities), the median change in the IWDAQ Composite Score was −4.0 for all subgroups of participants with BMI ≥27 to <35 (*n* = 49), ≥35 to <40 (*n* = 43), and ≥40 (*n* = 26).

**TABLE 5:** Descriptive statistics for change in the IWDAQ composite score from baseline to week 68 by the primary and supportive anchors.

Anchor	*n*	Mean (SD)	Median	95% CI
Primary anchor				
One‐category improvement on PGI‐S desired activities	118	−3.73 (2.59)	−4.00	−4.20, −3.26
Improved from ‘a little’ to ‘not at all’	77	−3.79 (2.27)	−4.00	−4.31, −3.28
Improved from ‘a moderate amount’ to ‘a little’	31	−3.61 (2.65)	−4.00	−4.59, −2.64
Improved from ‘a great deal’ to ‘a moderate amount’	9	−3.78 (4.74)	−5.00	−7.42, −0.14
Improved from ‘an extreme amount’ ‘a great deal’	1	−2.00 (−)	−2.00	−, −
Secondary anchors				
Two‐category improvement on PGI‐S Desired Activities	48	−5.54 (2.13)	−6.00	−6.16, −4.92
One‐category improvement on PGI‐S Necessary Activities	128	−3.86 (2.48)	−4.00	−4.29, −3.43
Two‐category improvement on PGI‐S Necessary Activities	35	−5.94 (2.25)	−6.00	−6.72, −5.17
’Somewhat less limited now’ on PGI‐C Desired Activities	45	−3.69 (2.42)	−3.00	−4.42, −2.96
’Somewhat less limited now’ on PGI‐C Necessary Activities	47	−3.23 (2.64)	−3.00	−4.01, −2.46

*Note*: Negative change scores indicate improvement.

Abbreviations: CI, confidence interval; IWDAQ, Impact of Weight on Daily Activities Questionnaire; PGI‐C, Patient Global Impression of Change; PGI‐S, Patient Global Impression of Status; SD, standard deviation.

The 0.5 SD for the baseline IWDAQ Composite Score was 1.37, and the standard error of measurement ranged from 1.17 (based on an ICC of 0.82) to 1.31 (based on an ICC of 0.77). As expected, the distribution‐based statistics are considerably lower than the anchor‐based estimates, thus supporting these estimates as reflecting change that is above measurement error. Taken together, the anchor‐based results indicate that a 4‐point improvement in the IWDAQ Composite Score is a reasonable threshold for meaningful improvement from the patient perspective.

## 4 DISCUSSION

The IWDAQ was developed to be a personalized assessment of the limitations in daily activities due to excess weight that matter most to individual patients, and its content validity was established through qualitative research.^4^ OASIS 1 participants selected a wide range of activities that they most wanted to see improve with weight loss, and across the 366 participants in the analysis sample, there were 152 different combinations of activities selected. There was no evidence of differences in scores between limitations in relatively basic activities (e.g., getting up from the floor or ground) and limitations in activities that were more physically demanding (e.g., strenuous exercise or activities), suggesting that participants selected activities according to their own ability level, as was intended in the design of the IWDAQ. Such findings are consistent with those from qualitative interviews, in which the nature of the activities currently limited by weight and considered important for improvement varied by BMI.^4^

The target concept for the IWDAQ is individualized, such that a person's score on the measure is a function of the activities selected as well as the limitations experienced. Consequently, the scale, rather than reflecting a single underlying construct of functional ability (i.e., a reflective measurement model), is intended as a composite of the limitations the individual experiences in the three activities that are most important to them (i.e., a composite measurement model).^29^ For clinical outcome assessments based on a composite measurement model, it is not a requirement for the constituent items to be associated with one another because it is not assumed that they reflect or are caused by a single underlying concept. This is consistent with the different types of activities included in the IWDAQ, which can potentially be affected by different aspects of having excess weight, such as physical limitations and/or psychosocial impacts. The diversity in the activities selected, together with the variability in the patterns of associations across the activities (both with each other and with external measures), suggests that a composite measurement model is most appropriate for the IWDAQ. Based on the results of a missing score analysis and consistent with this measurement model, it is recommended that the IWDAQ Composite Score be computed only if there are no missing data (i.e., ratings for limitations in all three activities must be completed).

While the patterns of correlations between the IWDAQ Composite Score and scores on most PRO measures were as hypothesized, the association between the IWDAQ composite score and the SF‐36v2 acute MCS score was lower than hypothesized, and weak associations were found between the SF‐36v2 acute Role Limitations–Emotional and Mental Health subscale scores, potentially suggesting that changes in activity limitations are not closely related to changes in general mental health in this population. Other studies have similarly reported that overweight/obesity did not impact MCS scores, despite impairments in physical activities.^30^ Nonetheless, while there was a tendency for some physical activity scores to correlate more closely with physical‐related scores than with psychosocial‐related scores on the IWQOL‐Lite‐CT and SF‐36v2 acute (and vice versa for some social activity scores), this was not consistently the case. For example, correlations between the IWDAQ composite score and the SF‐36v2 Social Functioning and Vitality subscales were moderate in magnitude. This may be because patients with obesity often experience psychosocial detriments that can cause them to avoid public places and group activities.^31^ Likewise, patients with obesity are at risk for lowered energy, which can impact household or work activities.^32^ This suggests that in the context of the IWDAQ, activity scores are related to both physical and psychosocial aspects of weight, thus supporting the computation of a single composite score.

Analyses to estimate MWPC thresholds for the IWDAQ Composite Score used a one‐category improvement on the PGIS Desired Activities (defined as the primary anchor). The results indicated that a 4‐point improvement on the IWDAQ Composite Score reflects meaningful improvement from the patient perspective. The consistency of the obtained estimates across participants with different levels of baseline severity and BMI indicates that the proposed MWPC threshold will have broad applicability. These results are consistent with findings from the qualitative interviews that were conducted with patients with obesity as part of the development of the IWDAQ.^4^ Specifically, during these interviews, the majority of participants (>80%) indicated that an improvement of one category on the PGIS response scale would correspond to a meaningful change. In addition, most participants (>75%) reported that a one‐category improvement on the IWDAQ response scale (which corresponds to a 3‐point improvement across three selected activities) would represent a meaningful change in their activity limitation. Thus, the qualitative data support both the selection of the primary anchor for meaningful change and the proposed MWPC threshold for the IWDAQ Composite Score.

Some limitations of this evaluation must be considered. First, while this study used the OASIS 1 trial population, over 70% of participants were female and were White, which may affect the generalizability of this study's findings. Second, while individuals select the IWDAQ activities that are most relevant to them at the initiation of weight‐loss treatment, whether they might find another set of activities more relevant at a follow‐up timepoint warrants consideration. Also, the number of observations for many of the activities, combined with the wide range of activity combinations selected, precluded a robust evaluation of the patterns of associations among the activities and with supporting measures. Further evaluations using data from additional studies would be valuable to extend this body of evidence. For example, data from larger samples would allow additional exploration of the factors affecting activity selection, as well as the patterns of associations between activity scores and the underlying measurement structure (e.g., using structural equation modelling approaches), thus strengthening the evidence supporting the measure.

## 5 CONCLUSIONS

The findings from this psychometric evaluation—using data pooled across treatment arms from the semaglutide OASIS 1 phase 3a trial—demonstrate that the IWDAQ is a reliable and valid personalized measure of activity limitations due to excess weight and provide support for the measurement properties of the IWDAQ. Building on the rigorous qualitative development, the adaptive design maximises the patient‐centricity of the measure, thus providing a unique and important perspective to complement existing measures of functioning^8^, ^9^, ^10^, ^11^ in the context of weight‐management clinical trials. The IWDAQ has the potential to be a key component of a comprehensive, patient‐focused outcomes measurement strategy in weight‐management clinical trials. Specifically, its adaptive design can complement outcomes data gathered using traditional PRO measures. Further, because the measure focuses on limitations in daily activities that are important to and selected by the individual, longitudinal implementation of the IWDAQ more closely mirrors ongoing interactions between patients and their healthcare professionals in clinical practice. Given the individualized design and the associated composite framework, the value of the IWDAQ lies predominantly in the assessment of change over time, and the fact that patients select the most important concepts to be assessed longitudinally offers a unique advantage in terms of item relevance.

## FUNDING INFORMATION

Novo Nordisk A/S provided the financial support for the study. RTI Health Solutions, an independent nonprofit research organization, received funding under a research contract with Novo Nordisk A/S to conduct this study and provide publication support in the form of manuscript writing, styling, and submission.

## CONFLICT OF INTEREST STATEMENT

This study was conducted under a research contract between RTI Health Solutions and Novo Nordisk A/S and was funded by Novo Nordisk A/S. Diane Whalley, Stuart Yarr, and Sheri E. Fehnel are salaried employees of RTI Health Solutions and did not receive any payment for their contributions to this publication. Lisa von Huth Smith and Jonathan Comins are salaried employees of Novo Nordisk A/S and hold Novo Nordisk stock.

## Supporting information


**Data S1.** Supporting Information.
