# Supplementary material for: Impact of weight on daily activities questionnaire in patients with overweight or obesity: Psychometric evaluation using data from the OASIS 1 trial
Source: Clin Obes. 2025 Jun 1;15(4):e70015. doi: 10.1111/cob.70015 (PMC12289406; doi:10.1111/cob.70015)
Supplement: Supplementary file 1 — Data S1. Supporting Information. [file COB-15-e70015-s001.pdf]

Impact of Weight on Daily Activities Questionnaire (IWDAQ) in Patients With Overweight or Obesity: Psychometric Evaluation Using Data From the OASIS 1 Trial

Authors:

Lisa von Huth Smith; Novo Nordisk A/S, Søborg, Denmark

Diane Whalley; Patient-Centered Outcomes Assessment, RTI Health Solutions, Towers Business Park, Manchester, United Kingdom

Stuart Yarr; Patient-Centered Outcomes Assessment, RTI Health Solutions, Towers Business Park, Manchester, United Kingdom

Jonathan Comins; Novo Nordisk A/S, Søborg, Denmark

Sheri E. Fehnel; Patient-Centered Outcomes Assessment, RTI Health Solutions, Research Triangle Park, NC, United States

Corresponding Author: Diane Whalley (Address: RTI Health Solutions, Towers Business Park, Manchester, United Kingdom; Email: dwhalley@rti.org)

SUPPORTING INFORMATION

Table S1: Overview of PRO Measures Used in the Psychometric Evaluation of the IWDAQ

Measure	Description and scoring	Recall period	Data used in the evaluation
IWDAQ	▪ Participants select which 3 activities they would most want to see improve with weight loss and rate the current limitation they experience in each of these 3 activities. At each follow-up assessment, participants then rate the degree of limitation with the same 3 activities.<br>– Bathing or showering<br>– Dressing or undressing<br>– Getting up from a chair<br>– Getting up from the floor or ground<br>– Sleeping<br>– Moving around the home<br>– Walking short distances<br>– Household tasks<br>– Shopping<br>– Playing with or taking care of children<br>– Socializing with people you know well<br>– Going to parties<br>– Dating or developing romantic relationships<br>– Sexual activity<br>– Outdoor tasks<br>– Physical activities at work<br>– Moderate exercise<br>– Strenuous exercise<br>▪ 5-point response scale:<br>– Not at all (scored 1)<br>– A little (scored 2)<br>– A moderate amount (scored 3)<br>– A great deal (scored 4)<br>– An extreme amount (scored 5)	Currently	Baseline, week 52, week 68, and change from baseline to week 68
PGI-S Desired Activities	▪ Single item: “How much does your weight currently limit your ability to do the things you would like to do on a regular basis?”<br>▪ 5-point scale:<br>– Not at all (scored 1)<br>– A little (scored 2)<br>– A moderate amount (scored 3)<br>– A great deal (scored 4)<br>– An extreme amount (scored 5)	Currently	Baseline, week 52, week 68, and change from baseline to week 68

Measure	Description and scoring	Recall period	Data used in the evaluation
PGI-S Necessary Activities	▪ Single item: "How much does your weight currently limit your ability to do the things you need to do on a regular basis?"<br>▪ 5-point scale:<br>– Not at all (scored 1)<br>– A little (scored 2)<br>– A moderate amount (scored 3)<br>– A great deal (scored 4)<br>– An extreme amount (scored 5)	Currently	Baseline, week 52, week 68, and change from baseline to week 68
PGI-S Physical Function	▪ Single item: "How would you rate your physical functioning (mobility and ability to do physical activities) at your current weight?"<br>▪ 5-point scale:<br>– Poor (scored 1)<br>– Fair (scored 2)<br>– Good (scored 3)<br>– Very good (scored 4)<br>– Excellent (scored 5)	Currently	Baseline, week 52, week 68, and change from baseline to week 68
PGI-S Mental Health	▪ Single item: "How would you rate how you feel mentally (emotions, self-confidence) at your current weight?"<br>▪ 5-point scale:<br>– Poor (scored 1)<br>– Fair (scored 2)<br>– Good (scored 3)<br>– Very good (scored 4)<br>– Excellent (scored 5)	Currently	Baseline, week 52, week 68, and change from baseline to week 68
PGI-C Desired Activities	▪ Single item: "Compared to the start of the study, how much does your weight currently limit your ability to do the things you would like to do on a regular basis?"<br>▪ 7-point scale:<br>– Much less limited now (scored 1)<br>– Somewhat less limited now (scored 2)<br>– A little less limited now (scored 3)<br>– No change (scored 4)<br>– A little more limited now (scored 5)<br>– Somewhat more limited now (scored 6)<br>– Much more limited now (scored 7)	Compared to the start of the study	Week 68

Measure	Description and scoring	Recall period	Data used in the evaluation
PGI-C Necessary Activities	▪ Single item: "Compared to the start of the study, how much does your weight currently limit your ability to do the things you need to do on a regular basis?"<br>▪ 7-point scale:<br>– Much less limited now (scored 1)<br>– Somewhat less limited now (scored 2)<br>– A little less limited now (scored 3)<br>– No change (scored 4)<br>– A little more limited now (scored 5)<br>– Somewhat more limited now (scored 6)<br>– Much more limited now (scored 7)	Compared to the start of the study	Week 68
PGI-C Physical Function	▪ Single item: "How would you rate your physical functioning (mobility and ability to do physical activities) at your current weight as compared to the beginning of the study?"<br>▪ 7-point scale:<br>– Much better (scored 1)<br>– Moderately better (scored 2)<br>– A little better (scored 3)<br>– No difference (scored 4)<br>– A little worse (scored 5)<br>– Moderately worse (scored 6)<br>– Much worse (scored 7)	Compared to the beginning of the study	Week 68
PGI-C Mental Health	▪ Single item: "How would you rate how you feel (emotions, self-confidence) at your current weight as compared to the beginning of the study?"<br>▪ 7-point scale:<br>– Much better (scored 1)<br>– Moderately better (scored 2)<br>– A little better (scored 3)<br>– No difference (scored 4)<br>– A little worse (scored 5)<br>– Moderately worse (scored 6)<br>– Much worse (scored 7)	Compared to the beginning of the study	Week 68

Measure	Description and scoring	Recall period	Data used in the evaluation
IWQOL-Lite-CT[8,10]	▪ 20 items assessing the impact of weight changes on patients' functioning within the context of clinical trials. Each item used a 5-point response scale:<br>– Never or not at all true (scored 1)<br>– Rarely or a little true (scored 2)<br>– Sometimes or moderately true (scored 3)<br>– Usually or mostly true (scored 4)<br>– Always or completely true (scored 5)<br>▪ Total score and 3 composite scores<br>– Physical (7 items)<br>– Physical Function (5 items)<br>– Psychosocial (13 items)<br>▪ Transformed total score and 3 composite scores range 0-100; higher scores indicate better status	Currently	Baseline, week 68, and change from baseline to week 68
SF-36v2 acute[11]	▪ 36 items assessing generic health status<br>▪ 8 subscales and 2 summary scores<br>– Physical Functioning (10 items)<br>– Role Limitations–Physical (4 items)<br>– Bodily Pain (2 items)<br>– Social Functioning (2 items)<br>– Mental Health (5 items)<br>– Role Limitations–Emotional (3 items)<br>– Vitality (4 items)<br>– General Health (5 items)<br>– Physical Component Summary<br>– Mental Component Summary<br>▪ *T* scores based on US population normative data with a mean of 50 and SD of 10; higher scores indicate better status	Past 1 week	Baseline, week 68, and change from baseline to week 68

IWQOL-Lite-CT = Impact of Weight on Quality of Life–Lite Clinical Trials Version; PGI-C = Patient Global Impression of Change; PGI-S = Patient Global Impression of Status; SD = standard deviation; SF-36v2 = Short Form Health Survey–36, version 2; US = United States.

Table S2: Descriptive Statistics for Measures Used in the Psychometric Analysis of the IWDAQ

Supporting measure	Baseline	Week 68
IWQOL-Lite-CT Physical mean (SD); median [min, max]; n	61.87 (21.68); 64.29 [0.0, 100.0]; 366	72.00 (21.52); 76.79 [7.1, 100.0]; 320
IWQOL-Lite-CT Physical Function mean (SD); median [min, max]; n	62.99 (22.56); 65.00 [0.0, 100.0]; 366	74.20 (22.65); 80.00 [5.0, 100.0]; 320
IWQOL-Lite-CT Psychosocial mean (SD); median [min, max]; n	59.96 (23.27); 62.50 [0.0, 100.0]; 366	73.94 (21.51); 79.17 [8.3, 100.0]; 320
IWQOL-Lite-CT Total mean (SD); median [min, max]; n	60.66 (20.70); 61.84 [3.9, 100.0]; 366	73.22 (19.63); 76.32 [11.8, 100.0]; 320
SF-36v2 acute PCS mean (SD); median [min, max]; n	55.64 (5.68); 56.71 [27.3, 71.9]; 366	54.52 (6.63); 56.16 [19.3, 69.0]; 320
SF-36v2 acute MCS mean (SD); median [min, max]; n	51.27 (7.31); 53.22 [21.5, 62.6]; 366	52.65 (7.14); 54.88 [25.7, 70.9]; 320
SF-36v2 acute Physical Functioning mean (SD); median [min, max]; n	51.39 (6.89); 53.74 [24.8, 57.6]; 366	52.61 (6.62); 55.67 [21.0, 57.6]; 320
SF-36v2 acute Role Limitations–Physical mean (SD); median [min, max]; n	53.10 (6.17); 57.12 [26.3, 57.1]; 366	53.94 (5.75); 57.12 [28.5, 57.1]; 320
SF-36v2 acute Bodily Pain mean (SD); median [min, max]; n	52.01 (8.28); 54.55 [30.1, 60.9]; 366	52.62 (8.38); 54.55 [21.4, 60.9]; 320
SF-36v2 acute General Health mean (SD); median [min, max]; n	52.82 (7.24); 54.82 [30.1, 65.4]; 366	53.23 (7.55); 55.26 [27.9, 65.4]; 320
SF-36v2 acute Vitality mean (SD); median [min, max]; n	55.28 (7.74); 55.54 [31.1, 69.2]; 366	55.27 (8.26); 55.54 [28.3, 69.2]; 320
SF-36v2 acute Social Functioning mean (SD); median [min, max]; n	54.38 (5.42); 56.74 [17.2, 56.7]; 366	54.22 (5.49); 56.74 [27.1, 56.7]; 320
SF-36v2 acute Role Limitations–Emotional mean (SD); median [min, max]; n	53.68 (4.57); 55.64 [25.1, 55.6]; 366	52.96 (5.87); 55.64 [28.9, 55.6]; 320
SF-36v2 acute Mental Health mean (SD); median [min, max]; n	55.28 (5.65); 57.72 [30.5, 62.7]; 366	54.64 (6.56); 55.24 [25.5, 62.7]; 320
BMI (kg/m^2) mean (SD); median [min, max]; n	37.40 (6.10); 36.17 [27.0, 61.6]; 366	33.71 (7.06); 32.99 [21.4, 61.5]; 328
Body weight (kg) mean (SD); median [min, max]; n	104.69 (20.41); 100.75 [66.0, 186.5]; 366	94.72 (22.65); 92.35 [54.9, 173.1]; 328
PGI-S Desired Activities mean (SD); median [min, max]; n	2.39 (1.05); 2.00 [1.0, 5.0]; 366	1.66 (0.89); 1.00 [1.0, 5.0]; 319
PGI-S Necessary Activities mean (SD); median [min, max]; n	1.97 (0.89); 2.00 [1.0, 5.0]; 366	1.37 (0.68); 1.00 [1.0, 4.0]; 320
PGI-S Physical Function mean (SD); median [min, max]; n	2.64 (1.02); 3.00 [1.0, 5.0]; 365	3.24 (1.03); 3.00 [1.0, 5.0]; 320
PGI-S Mental Health mean (SD); median [min, max]; n	2.86 (1.01); 3.00 [1.0, 5.0]; 366	3.47 (1.05); 4.00 [1.0, 5.0]; 320
PGI-C Desired Activities mean (SD); median [min, max]; n	-	2.47 (1.38); 2.00 [1.0, 7.0]; 319

Supporting measure	Baseline	Week 68
PGI-C Necessary Activities mean (SD); median [min, max]; n	-	2.43 (1.36); 2.00 [1.0, 6.0]; 320
PGI-C Physical Function mean (SD); median [min, max]; n	-	2.31 (1.29); 2.00 [1.0, 6.0];320
PGI-C Mental Health mean (SD); median [min, max]; n	-	2.43 (1.33); 2.00 [1.0, 7.0]; 320

BMI = body mass index; IWQOL-Lite-CT = Impact of Weight on Quality of Life–Lite Clinical Trials Version; MCS = Mental Component Summary; PCS = Physical Component Summary; PGI-C = Patient Global Impression of Change; PGI-S = Patient Global Impression of Status; SD = standard deviation; SF-36v2 = Short Form Health Survey-36, version 2.

Table S3: Descriptive Statistics and Response Distributions for IWDAQ Activity Scores at Baseline and Week 68

IWDAQ activity	n (%)[a] of participants selecting the activity	Mean (SD) [median] score	Response: n (%)[b]				
			Not at all	A little	A moder-ate amount	A great deal	An extreme amount
**Baseline**							
1. Bathing or showering	7 (1.9)	2.1 (0.7) [2.0]	1 (14.3)	4 (57.1)	2 (28.6)	0 (0.0)	0 (0.0)
2. Getting dressed or undressed	26 (7.1)	3.0 (1.0) [3.0]	2 (7.7)	5 (19.2)	11 (42.3)	6 (23.1)	2 (7.7)
3. Getting up from a low chair	31 (8.5)	3.1 (1.1) [3.0]	0 (0.0)	11 (35.5)	9 (29.0)	7 (22.6)	4 (12.9)
4. Getting up from the floor or ground	111 (30.3)	3.2 (1.0) [3.0]	1 (0.9)	27 (24.3)	39 (35.1)	34 (30.6)	10 (9.0)
5. Getting a good night’s sleep	109 (29.8)	2.6 (1.0) [2.0]	11 (10.1)	47 (43.1)	27 (24.8)	20 (18.3)	4 (3.7)
6. Moving around inside home	11 (3.0)	3.0 (1.3) [3.0]	1 (9.1)	3 (27.3)	4 (36.4)	1 (9.1)	2 (18.2)
7. Walking short distances	17 (4.6)	2.6 (1.3) [2.0]	4 (23.5)	5 (29.4)	2 (11.8)	5 (29.4)	1 (5.9)
8. Household tasks	16 (4.4)	2.9 (1.0) [3.0]	0 (0.0)	7 (43.8)	5 (31.3)	3 (18.8)	1 (6.3)
9. Shopping for groceries	9 (2.5)	2.4 (1.5) [3.0]	4 (44.4)	0 (0.0)	3 (33.3)	1 (11.1)	1 (11.1)
10. Activities with children	62 (16.9)	2.7 (0.8) [3.0]	2 (3.2)	25 (40.3)	26 (41.9)	8 (12.9)	1 (1.6)
11. Socializing with people know well	32 (8.7)	2.2 (1.2) [2.0]	10 (31.3)	11 (34.4)	7 (21.9)	2 (6.3)	2 (6.3)
12. Socializing with people don’t know	43 (11.7)	2.9 (1.0) [3.0]	3 (7.0)	11 (25.6)	18 (41.9)	8 (18.6)	3 (7.0)
13. Developing romantic relationships	41 (11.2)	3.2 (1.2) [3.0]	4 (9.8)	8 (19.5)	12 (29.3)	10 (24.4)	7 (17.1)
14. Sexual activity	66 (18.0)	3.2 (1.0) [3.0]	4 (6.1)	12 (18.2)	23 (34.8)	21 (31.8)	6 (9.1)
15. Outdoor tasks	50 (13.7)	2.5 (0.9) [2.5]	6 (12.0)	19 (38.0)	18 (36.0)	6 (12.0)	1 (2.0)
16. Physical activities at work	74 (20.2)	2.4 (0.9) [2.0]	15 (20.3)	25 (33.8)	25 (33.8)	9 (12.2)	0 (0.0)
17. Moderate exercise or activities	205 (56.0)	2.9 (1.0) [3.0]	17 (8.3)	57 (27.8)	70 (34.1)	50 (24.4)	11 (5.4)
18. Strenuous exercise or activities	188 (51.4)	3.2 (1.2) [3.0]	12 (6.4)	44 (23.4)	47 (25.0)	56 (29.8)	29 (15.4)
**Week 68**							
1. Bathing or showering	5 (1.4)	1.2 (0.4) [1.0]	4 (80.0)	1 (20.0)	0 (0.0)	0 (0.0)	0 (0.0)
2. Getting dressed or undressed	19 (5.2)	1.9 (1.4) [1.0]	11 (57.9)	4 (21.1)	1 (5.3)	1 (5.3)	2 (10.5)
3. Getting up from a low chair	26 (7.1)	1.8 (1.0) [2.0]	12 (46.2)	9 (34.6)	4 (15.4)	0 (0.0)	1 (3.8)
4. Getting up from the floor or ground	102 (27.9)	2.2 (1.2) [2.0]	35 (34.3)	33 (32.4)	16 (15.7)	14 (13.7)	4 (3.9)
5. Getting a good night’s sleep	90 (24.6)	1.6 (0.8) [1.0]	52 (57.8)	28 (31.1)	6 (6.7)	4 (4.4)	0 (0.0)
6. Moving around inside home	9 (2.5)	1.9 (1.5) [1.0]	6 (66.7)	1 (11.1)	0 (0.0)	1 (11.1)	1 (11.1)
7. Walking short distances	11 (3.0)	1.4 (0.7) [1.0]	8 (72.7)	2 (18.2)	1 (9.1)	0 (0.0)	0 (0.0)
8. Household tasks	15 (4.1)	1.6 (0.6) [2.0]	7 (46.7)	7 (46.7)	1 (6.7)	0 (0.0)	0 (0.0)

IWDAQ activity	n (%)[a] of participants selecting the activity	Mean (SD) [median] score	Response: n (%)[b]				
			Not at all	A little	A moder-ate amount	A great deal	An extreme amount
9. Shopping for groceries	6 (1.6)	1.5 (0.8) [1.0]	4 (66.7)	1 (16.7)	1 (16.7)	0 (0.0)	0 (0.0)
10. Activities with children	58 (15.8)	1.8 (0.9) [2.0]	24 (41.4)	24 (41.4)	6 (10.3)	4 (6.9)	0 (0.0)
11. Socializing with people know well	30 (8.2)	1.5 (0.9) [1.0]	22 (73.3)	3 (10.0)	3 (10.0)	2 (6.7)	0 (0.0)
12. Socializing with people don't know	36 (9.8)	1.6 (0.8) [1.0]	20 (55.6)	12 (33.3)	3 (8.3)	1 (2.8)	0 (0.0)
13. Developing romantic relationships	31 (8.5)	1.8 (1.2) [1.0]	17 (54.8)	9 (29.0)	0 (0.0)	4 (12.9)	1 (3.2)
14. Sexual activity	60 (16.4)	1.7 (0.9) [1.0]	32 (53.3)	20 (33.3)	4 (6.7)	3 (5.0)	1 (1.7)
15. Outdoor tasks	47 (12.8)	1.6 (1.0) [1.0]	31 (66.0)	6 (12.8)	8 (17.0)	1 (2.1)	1 (2.1)
16. Physical activities at work	70 (19.1)	1.6 (0.9) [1.0]	39 (55.7)	23 (32.9)	5 (7.1)	2 (2.9)	1 (1.4)
17. Moderate exercise or activities	181 (49.5)	1.8 (1.0) [1.0]	99 (54.7)	45 (24.9)	21 (11.6)	12 (6.6)	4 (2.2)
18. Strenuous exercise or activities	164 (44.8)	2.0 (1.1) [2.0]	68 (41.5)	50 (30.5)	24 (14.6)	19 (11.6)	3 (1.8)

IWDAQ = Impact of Weight on Daily Activities Questionnaire; SD = standard deviation.

Note: Each participant provided ratings for the same 3 IWDAQ activities at all timepoints.

[a] Percentages are calculated out of the number of participants who completed the IWDAQ at the respective timepoint.

[b] Percentages are calculated out of the number of participants who selected each activity at baseline and completed the IWDAQ at the respective timepoint.

Table S4: Test-Retest Reliability Coefficients for IWDAQ Activity Scores at Weeks 52 and 68

	Weighted kappa (95% CI), n		
**IWDAQ score**	**All participants**	**Participants with < 5% change in body weight and no change on PGI-S Desired Activities**	**Participants with < 5% change in body weight and no change on PGI-S Necessary Activities**
1. Bathing or showering	–[a]	–[a]	–[a]
2. Getting dressed or undressed	0.76 (0.45, 1.00), 18	–[a]	0.58 (0.27, 0.89), 12
3. Getting up from a low chair	0.53 (0.32, 0.74), 24	0.51 (0.10, 0.92), 14	0.57 (0.22, 0.92), 15
4. Getting up from the floor or ground	0.69 (0.55, 0.83), 98	0.76 (0.61, 0.90), 54	0.70 (0.48, 0.91), 60
5. Getting a good night's sleep	0.62 (0.41, 0.83), 81	0.57 (0.27, 0.86), 52	0.44 (0.13, 0.74), 52
6. Moving around inside home	–[a]	–[a]	–[a]
7. Walking short distances	–[a]	–[a]	–[a]
8. Household tasks	0.69 (0.44, 0.95), 13	–[a]	–[a]
9. Shopping for groceries	–[a]	–[a]	–[a]
10. Activities with children	0.60 (0.35, 0.84), 56	0.55 (0.17, 0.93), 35	0.57 (0.25, 0.89), 42
11. Socializing with people know well	0.31 (−0.13, 0.74), 25	−0.12 (−0.28, 0.03), 18	0.45 (0.02, 0.89), 16
12. Socializing with people don't know	0.39 (0.04, 0.73), 35	0.59 (0.26, 0.92), 21	0.57 (0.18, 0.95), 22
13. Developing romantic relationships	0.86 (0.77, 0.95), 30	0.87 (0.75, 1.00), 16	0.86 (0.70, 1.00), 17
14. Sexual activity	0.65 (0.46, 0.85), 55	0.59 (0.30, 0.87), 34	0.48 (0.21, 0.74), 33
15. Outdoor tasks	0.86 (0.76, 0.95), 46	0.88 (0.79, 0.98), 27	0.82 (0.63, 1.00), 30
16. Physical activities at work	0.62 (0.36, 0.88), 68	0.54 (0.12, 0.95), 39	0.69 (0.52, 0.87), 41
17. Moderate exercise or activities	0.74 (0.67, 0.82), 173	0.74 (0.61, 0.87), 88	0.66 (0.54, 0.79), 105
18. Strenuous exercise or activities	0.77 (0.70, 0.84), 156	0.84 (0.77, 0.90), 99	0.80 (0.72, 0.88), 105

CI = confidence interval; IWDAQ = Impact of Weight on Daily Activities Questionnaire; PGI-S = Patient Global Impression of Status.

Notes: Each participant provided ratings for the same 3 IWDAQ activities at all timepoints. Guideline for level of agreement from weighted kappa coefficients:[25] < 0 = poor, 0-0.20 = slight, 0.21-0.40 = fair, 0.41-0.60 = moderate, 0.61-0.80 = substantial, and > 0.80 = almost perfect agreement.

[a] Weighted kappa coefficient was not computed due to sample size < 10.

Figure S1: Empirical Cumulative Distribution Function Plot for Change in the IWDAQ Composite Score by Change in PGI-S Desired Activities From Baseline to Week 68

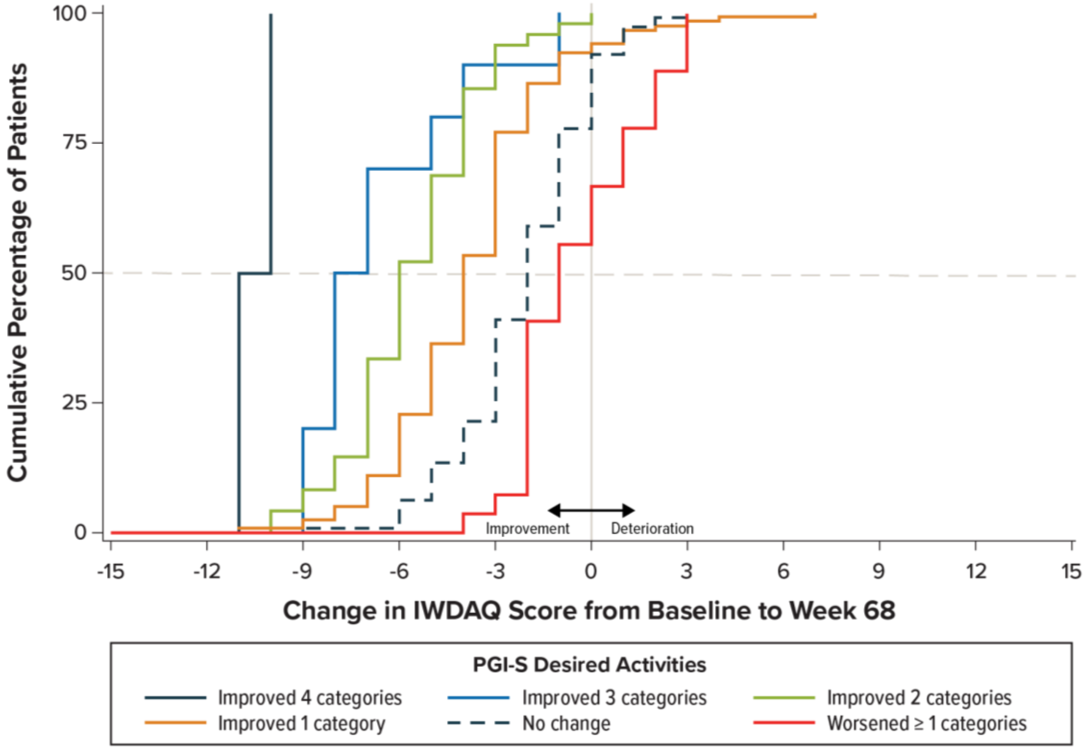

IWDAQ = Impact of Weight on Daily Activities Questionnaire; PGI-S = Patient Global Impression of Status.
Note: Change is from baseline to week 68; negative change scores indicate improvement.
